# Does risk for ovarian malignancy algorithm excel human epididymis protein 4 and ca125 in predicting epithelial ovarian cancer: A meta-analysis

**DOI:** 10.1186/1471-2407-12-258

**Published:** 2012-06-19

**Authors:** Fake Li, Ruxiu Tie, Kai Chang, Feng Wang, Shaoli Deng, Weiping Lu, Lili Yu, Ming Chen

**Affiliations:** 1 Department of Clinical Laboratory, Institute of Surgery Research, Daping Hospital, Third Military Medical University, ChongQing, Peoples Republic of China; 2 Department of Obstetrics and Gynecology, Institute of Surgery Research, Daping Hospital, Third Military Medical University, ChongQing, Peoples Republic of China

## Abstract

**Backgrounds:**

Risk for Ovarian Malignancy Algorithm (ROMA) and Human epididymis protein 4 (HE4) appear to be promising predictors for epithelial ovarian cancer (EOC), however, conflicting results exist in the diagnostic performance comparison among ROMA, HE4 and CA125.

**Methods:**

Remote databases (MEDLINE/PUBMED, EMBASE, Web of Science, Google Scholar, the Cochrane Library and ClinicalTrials.gov) and full texts bibliography were searched for relevant abstracts. All studies included were closely assessed with the QUADAS-2 (Quality Assessment of Diagnostic Accuracy Studies-2). EOC predictive value of ROMA was systematically evaluated, and comparison among the predictive performances of ROMA, HE4 and CA125 were conducted within the same population. Sensitivity, specificity, DOR (diagnostic odds ratio), LR ± (positive and negative likelihood ratio) and AUC (area under receiver operating characteristic-curve) were summarized with a bivariate model. Subgroup analysis and sensitivity analysis were used to explore the heterogeneity.

**Results:**

Data of 7792 tests were retrieved from 11 studies. The overall estimates of ROMA for EOC predicting were: sensitivity (0.89, 95% CI 0.84-0.93), specificity (0.83, 95% CI 0.77-0.88), and AUC (0.93, 95% CI 0.90-0.95). Comparison of EOC predictive value between HE4 and CA125 found, specificity: HE4 (0.93, 95% CI 0.87-0.96) > CA125 (0.84, 95% CI 0.76-0.90); AUC: CA125 (0.88, 95% CI 0.85-0.91) > HE4 (0.82, 95% CI 0.78-0.85). Comparison of OC predictive value between HE4 and CA125 found, AUC: CA125 (0.89, 95% CI 0.85-0.91) > HE4 (0.79, 95% CI 0.76-0.83). Comparison among the three tests for EOC prediction found, sensitivity: ROMA (0.86, 95%CI 0.81-0.91) > HE4 (0.80, 95% CI 0.73-0.85); specificity: HE4 (0.94, 95% CI 0.90-0.96) > ROMA (0.84, 95% CI 0.79-0.88) > CA125 (0.78, 95%CI 0.73-0.83).

**Conclusions:**

ROMA is helpful for distinguishing epithelial ovarian cancer from benign pelvic mass. HE4 is not better than CA125 either for EOC or OC prediction. ROMA is promising predictors of epithelial ovarian cancer to replace CA125, but its utilization requires further exploration.

## Background

Ovarian cancer is the leading cause of death from gynecologic cancers in the United States and the fifth-top cause of cancer death in women (Link 1). Non-specific clinical manifestation mainly hinders the early diagnosis of ovarian cancer[1]. Cancer antigen 125 (CA125) was the only FDA-approved biomarker for ovarian cancer before the year 2008. CA125 is indicated for use as an aid in the detection of residual ovarian carcinoma in patients who have undergone first-line therapy and would be considered for diagnostic second-look procedures. Although the CA125 serum level elevated in 80% of epithelial ovarian cancer (EOC) patients with advanced stage [2], it increased in only 50% of patients with stage I EOC [3]. In addition, CA125 serum levels elevate in various benign gynecological diseases (including endometriosis) [4], non-gynecologic malignancies [5]. Therefore, considerable efforts are underway to identify new serum biomarkers, alone or combining with CA125 to improve EOC detection [6,7].

With high-throughput technologies employed, a large number of new biomarkers have been discovered [8-10]. Human epididymis protein 4 (HE4) is among the most promising ones [11]. High levels of HE4 are found in the serum of patients with EOC, especially in serous and endometroid cancers [12]. Unlike CA125, HE4 doesn’t overexpress in endometriosis and other benign gynecological diseases [11]. And HE4, as an aid in monitoring recurrence or progressive disease in patients with epithelial ovarian cancer, has been the first biomarker for EOC after CA125 to be approved by the U.S. Food and Drug Administration (FDA) at the year of 2008. However, conflicts arise on the sensitivity of HE4 and CA125 [5,13-16].

Moore and colleagues [17] have explored a multianalytes assay named the Risk of Ovarian Malignancy Algorithm (ROMA™), which combines the results of HE4 EIA (enzyme immunoassay), ARCHITECT CA 125 II™ and menopausal status into a numerical score to predict malignancy when an ovarian mass was found clinically. Although ROMA™ has received clearance from the FDA of U.S. in September of the year 2011, the diagnostic accuracy of ROMA compared to CA125 and HE4 alone is still controversial [13,16-18]. Here we try to clarify conflicting results existing in the diagnostic accuracy of ROMA, and in the performance comparison among ROMA, HE4 and CA125.

## Methods

### Data sources and search strategy

We followed the Meta-analysis Of Observational Studies in Epidemiology (MOOSE)[19] and the Cochrane Handbook for Systematic Reviews of Diagnostic Test Accuracy (Link 2). MEDLINE (through PubMed interface), EMBASE, Web of Science, Google Scholar, the Cochrane Library and ClinicalTrials.gov (ended on 22^th^ December, 2011) were searched. Reference lists of articles identified were manually searched. Publication languages were not limited. The terminology for search was based on the standardized National Library of Medicine MeSH terms and free texts. The search strategies of all the databases were based on those of PubMed (Additional file 1: Table S1).

Two authors (RXT and WPL) independently screened the search results based on the titles and abstracts. The full text of selected articles were reviewed independently by another two authors (KC and LLY) to determine the inclusion. Disagreements were resolved by referring to a third author (MC).

### Inclusion criteria

Studies that investigated both serum HE4 and CA125 as diagnostic tests or calculated the ROMA algorithm were included if (1) they were cross-sectional studies; and (2) performed in the same population presenting pelvic mass; (3) all serum specimens were collected preoperatively; (4) all subjects with histological diagnostic information; (5) with sufficient data for reconstructing fourfold table.

Studies recruiting participants without presenting pelvis mass, with obviously error data or ROC curve analysis containing healthy person and case–control studies were excluded. Case–control studies were excluded, for these studies had a tendency of overestimating or underestimating the diagnostic performance of a test [20].

### Data extraction

The data extracted from each study included: author; year; country; design; recruitment; age; menopausal status; test methods (e.g. chemilumenesence immunoassay); number of patients; sensitivity; specificity and cut-off value. Four fold tables were reconstructed. Two reviewers (FKL and RXT) independently extracted the data for each study and referred to a third opinion (MC) when disagreements appeared. Important data that were not provided in the original studies were referred to their authors through Emails.

### Index tests and reference standard

Since the Risk of Ovarian Malignancy Algorithm (ROMA™) is a qualitative serum test that combines the results of HE4 EIA (enzyme immunometric assays), ARCHITECT CA 125 II™ and menopausal status into a numerical score. Index tests for HE4 and CA125 in this meta-analysis questions were specified as EIAs and chemilumenesence immunoassays respectively. ROMA algorithm is the following [17]:

(1) $$\text{Premenopausal}:\text{ predictive index }\left(\text{PI}\right)=-12.0+\left(2.38\times \text{LN}\left(\text{HE}4\right)\right)+\left(0.0626\times \text{LN}\left(\text{CA}125\right)\right)$$

(2) $$\text{Postmenopausal}:\text{ PI}=-8.09+\left(1.04\times \text{LN}\left(\text{HE}4\right)\right)+\left(0.732\times \text{LN}\left(\text{CA}125\right)\right)$$

(3) $$\text{Predicted probability}:\left(\text{PP}\right)=100\times \text{exp}\left(\text{PI}\right)/\left(1+\text{exp}\left(\text{PI}\right)\right)$$

Reference standard was based on outcomes of histopathological diagnosis. In all studies, ovarian cancer surgical stages were referred to criteria from FIGO (International Federation of Gynecology and Obsterics) [21] (Link 3). Early stage were defined as FIGO stages I & II, while advanced stage were FIGO stages III & IV.

### Methodological quality assessment

The methodological quality of each study was evaluated with QUADAS-2 (Quality Assessment of Diagnostic Accuracy Studies 2) [22] quality items. Overall scores were not helpful for interpreting study quality [23] and were avoided in studies evaluation by QUADAS-2 tool. Doubts were resolved by discussion. In the items of QUADAS-2, the blindness of index tests and reference test has been list, but not the blindness between index tests. So one item that focus on validity of this comparative question has been added in Risk of Bias part of Domain 2 (Index Test) in QUADAS-2 [22] as follows. “Were the results of index tests interpreted without knowledge of each other?” The answers (Yes, No or Unclear) of this question were considered to help assessing the Risk of Bias of including studies. According to the suggestion in Concerns Regarding Applicability part of Domain 2 (Index Test) in QUADAS-2 [22], variations in test technology, executing, or interpretation might affect estimates of the diagnostic accuracy of a test. If index test methods varied from those specified in the review question, concerns about applicability might exist.

Index tests for HE4 and CA125 in this meta-analysis questions were specified as EIAs and chemilumenesence immunoassays respectively. For tests of HE4, the chemilumenesence immunoassays were more sensitive than the specified EIAs, thus bias might be introduced into pooling of studies. And similarly, for CA125, EIA and RIA (radioimmunoassay) assays were less sensitive and steady than chemilumenesence immunoassays, so studies using either EIA or RIA will be considered as High Concern Regarding Applicability. The ROMA test employed the results from tests of CA125 and HE4 within the same study. So ROMA was considered as High Concern Regarding Applicability when either HE4 or CA125 test was evaluated as High Concern Regarding Applicability.

### Data analysis plan

The statistical analysis is based on the following steps: (1) qualitatively describing the findings; (2) searching for heterogeneity and threshold effect; (3) figuring out the sources of heterogeneity by subgroup analysis; (4) choosing appropriate model and pooling estimates statistically. Univariate [24] and bivariate model [25] were two choices for diagnostic meta-analysis. When a positive correlation existed between true positive rate (TPR) and false positive rate (FPR), the bivariate analysis model was more appropriate [26].

Heterogeneity of studies were shown with forest graphs and explored with I^2^ estimates [27]. The main advantage of I^2^ was inherent independence with the number of the studies included in the meta-analysis. I^2^ estimates below 25% were regarded as low risk of heterogeneity, between 25% and 50% as moderate heterogeneity, and 50% or higher as high heterogeneity. If there was a low level heterogeneity, univariate meta-analysis model was used (Meta-DiSc software version 1.4 [28]). If there was a moderate to high heterogeneity, Spearman correlation coefficients was explored. Positive Spearman correlation coefficients between Logit(TPR) and Logit(FPR) denoted the presence of threshold effects (Meta-DiSc software version 1.4). Then a bivariate model as well as HSROC (Hierarchical Summary Receiver Operator Characteristics) were estimated and plotted; if negative, summary estimates were pooled without HSROC [24,29]; and if zero, summary estimates were pooled the way same as low level heterogeneity.

Influence analysis reestimated the meta-analysis by omitting each study in turn (STATA version 10.0) to confirm the stability of our analysis model. Publication bias was investigated by Deek’s funnel plot as well as asymmetry test [30]. Subgroups were analyzed hierarchically by menopausal status, FIGO stages and concern of methods of index tests. In some studies, patients with low malignant potential tumors (LMP) or borderline tumors (BL) were classified into EOC group. And these studies were specifically analyzed as subgroup EOC (LMP/BL). Subgroups with less than four studies were analyzed with univariate model, because the bivariate model required 4 studies at least [26]. Summary estimates and 95% CIs (confidence intervals) for sensitivity, specificity, DOR, LR ± and AUC were calculated (STATA version 10.0 [31,32]). HSROC (Hierarchical summary receiver operating characteristic curves) plots were shown when appropriate. Comparisons between estimates of different tests were performed with z-test.

## Results

### Search results

**Figure 1:**
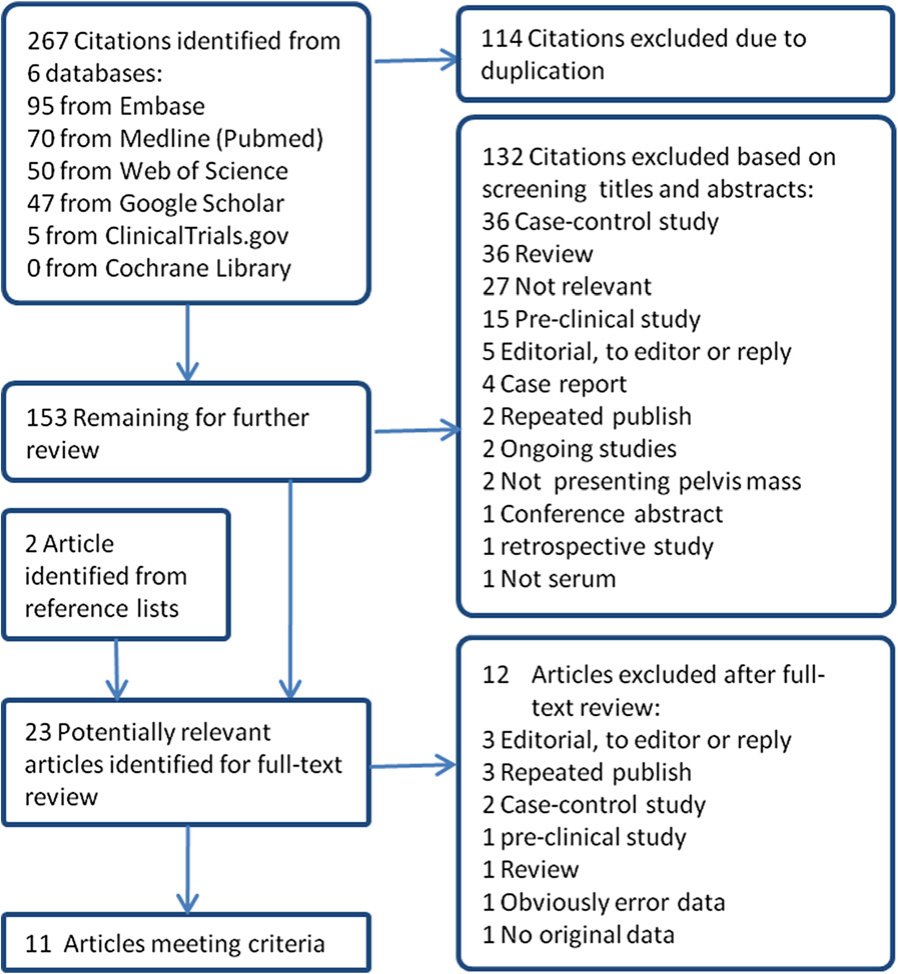
Flowchart of selection of eligible studies.

Of the 267 references identified from 6 databases, 11 articles [13-18,33-37] met the inclusion criteria and were included in meta-analysis (Figure1).

Characteristics of the included studies were summarized (Table1). 7792 tests from 2878 patients presenting pelvic mass at risk of ovarian cancer were retrieved. Of the 11 studies, 6 studies [15,17,18,34,36,37] enrolling 1547 patients investigated the performance of ROMA for EOC prediction. Five studies [16,33-36] with 883 patients compared the performance of HE4 and CA125 for OC prediction. Four studies [13,15,18,36] with 715 patients compared the performance of HE4 and CA125 for EOC prediction. And 3 studies [15,18,36] (482 patients) compared the performance among ROMA, HE4 and CA125 for EOC prediction. In all studies, the spectrum of patients was considered representative. All enrolled participants present pelvis mass of suspected ovarian origin, have never received any treatment before and plan to have a surgical intervention. The prevalence of proven ovarian cancer across all studies ranged from 7.86% to 63.1% (overall prevalence was 18.5% for EOC). The study of Holcomb and colleagues [14] had the lowest prevalence (7.86%) for only investigating the results of premenopausal women.

**Table 1:** Characteristics of studies included in the analysis

**Reference**	**Country**	**Study Design**	**Subjects (number)**	**Menopausal Condition PreM/PostM**	**Index Tests (Methods)**	**Cut-off Value**	**Proven cancer N (%)**
**Abdel-Azeez et al., 2010**	Egypt	Cross-section	65	18/47	HE4 (EIA); CA125 (ECLIA)	HE4: 72pM; CA125: 35U/mL	OC: 41 (63.1%)
**Bandiera et al., 2011**	Italy	Cross-section	419	134/284 (1 patient with unknown stage was not analyzed)	ROMA	ROMA: preM 7.4% postM 25.3%;	EOC: 114 (27.2%)
					HE4 (CMIA);	HE4: preM 70pM, postM 140pM;	
					CA125 (CMIA)	CA125: 35U/mL	
**Chang et al., 2011**	China	Cross-section	118	-	HE4 (EIA); CA125 (EIA)	HE4: 150pM; CA125: 35U/mL	OC: 52 (44.1%)
**Holcomb et al., 2011**	USA	Cross-section	229	229/-	HE4 (CMIA); CA125 (CMIA)	HE4: 70pM; CA125: 35U/mL	EOC: 18 (7.86%)
**Jacob et al., 2011**	Switzerland	Cross-section	160	84/76	ROMA	ROMA: 13.1%	EOC: 29 (18.1%)
					HE4 (ELISA); CA125 (ELISA)	HE4: 70pM; CA125: 35U/mL	OC: 56 (35%)
**Kim et al., 2011**	Korea	Cross-section	159	51/108	ROMA	ROMA: preM 7.6% postM 10.9%;	EOC: 72 (45.3%)
					HE4 (CMIA); CA125 (CMIA)	HE4: 70pM; CA125: 35 IU/mL	OC: 78 (49.1%)
**Montagnana et al., 2011**	Italy	Cross-section	104	51/53	ROMA	ROMA: preM12.5% postM14.4%;	EOC: 55 (52.9%)
					HE4 (EIA); CA125 (CLEIA)	HE4: 74.2pM; CA125: 35U/mL	
**Moore et al., 2008**	USA	Cross-section	233	-	HE4 (EIA); CA125 (RIA)	-	EOC: 67 (28.8%)
**Moore et al., 2009**	USA	Cross-section	531	248/283	ROMA	ROMA: preM 13.1% postM 27.7%	EOC: 129 (26.8%)
					HE4 (EIA); CA125 (CMIA)		OC: 154 (29.0%)
**Moore et al., 2011**	USA	Cross-section	472	255/217	ROMA	ROMA: preM 13.1%; postM 27.7%	EOC: 48 (10.2%)
					HE4 (ELISA); CA125 (CMIA)		
**Van Gorp et al., 2011**	Belgium	Cross-section	389	184/205	ROMA	ROMA: preM 12.5% postM 14.4%;	OC: 161 (41.4%)
					HE4 (EIA); CA125 (EIA)	HE4: 70pM;CA125: 35U/mL	

PreM: premenopause; PostM: postmenopause; ROMA: Risk of Ovarian Malignancy Algorithm; HE4: Human Epididymis Protein 4; CA125: Cancer Antigen 125; EOC: epithelial ovarian cancer; OC: ovarian cancer; EIA: enzyme immunoassay; CLEIA: chemilumenscence enzyme immunoassay; CMIA: chemiluminescent microparticle immunoassay; ECLIA: electrochemilumenscence immunoassay; RIA: radioimmunoassay; ELISA: enzyme-linked immunoadsorbent assay. -: not mentioned.

### Methods of index tests

**Figure 2:**
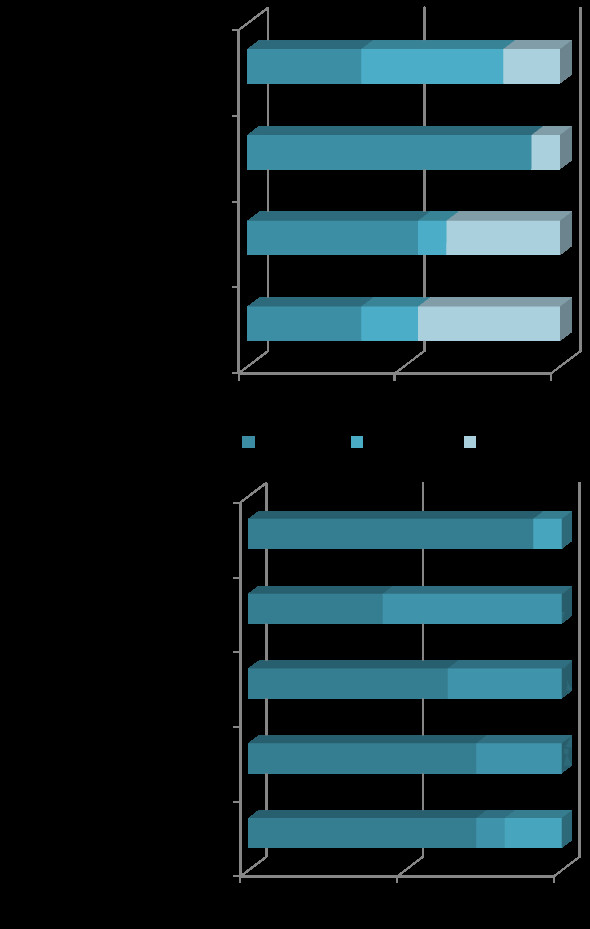
**Graph of QUADAS-2 quality items results.** Figure2**a**. Proportion of studies with low, high, or unclear risk of bias.Figure2**b**. Proportion of studies with low, high, or unclear Concerns Regarding Applicability. Three horizontal bars represented index tests HE4, CA125 and ROMA, respectively.

All of 11 including studies measured serum HE4 and CA125. For HE4 measurement, 8 studies [13,16-18,33,35-37] used EIA (enzyme immunoassay), the other 3 studies [14,15,34] employed CMIA (chemiluminescent microparticle immunoassay). For the measure of CA125, 5 studies [14,15,17,34,37] employed CMIA, 3 studies [16,35,36] with EIA, 3 studies [13,18,33] used RIA (radioimmunoassay), CLEIA (chemilumenscence enzyme immunoassay) and ECLIA (electrochemilumenscence immunoassay) respectively. CMIA, CLEIA and ECLIA belonged to chemilumenesence immunoassays, which were higher sensitive than EIA or RIA. According to Methodological quality assessment (the 4^th^ part of Methods section), HE4 tests with CMIA, CA125 tests with EIA and RIA were regarded as high Concern Regarding Applicability. The ROMA tests were considered as high Concern Regarding Applicability when either HE4 or CA125 test was evaluated as high Concern Regarding Applicability (Figure2).

### Methodological quality of all included studies

Quality of included studies was assessed by the QUADAS-2 tool (Figure2 & Table2). Within 9 [13,14,16-18,34-37] of 11 studies, the results interpretation of index tests (HE4/CA125) were blind with reference standard test (ROMA). The other 2 studies [15,33] were unclear. In 5 of the 11 studies [14,16,34-36] the results of index tests (HE4 and CA125) were interpreted without knowledge of each other. In the other 6 studies [13,15,17,18,33,37] the blindness was unclear. So when assessing the studies with the item “Could the conduct or interpretation of the index test have introduced bias?” in domain 2 of QUADAS-2, the results showed that 5 studies [14,16,34-36] were low risk of bias, 1 study [13] was high risk of bias and 5 studies [15,17,18,33,37] were unclear their risk of bias. Four [16-18,34] of the total 11 studies were considered as low risk of bias for the Patient Selection (Domain 1 of QUADAS-2) for their consecutive enrollment of patients; 2 studies [14,15] were regarded as high risk of bias and in the other 5 studies [13,33,35-37] the risk was unclear.

**Table 2:** QUADAS-2 quality items results

**Study ID**	**Risk of Bias**				**Concerns Regarding Applicability**				
	**PATIENT SELECTION**	**INDEX TEST**	**REFERENCE STANDARD**	**FLOW & TIMING**	**PATIENT SELECTION**	**INDEX TEST**			**REFERENCE STANDARD**
						**HE4**	**CA125**	**ROMA**	
Abdel-Azeez et al., 2010	?	?	?	?	☺	☺	☺	-	☺
Bandiera et al., 2011	☻	?	?	☻	☻	☻	☺	☻	☺
Chang et al., 2011	?	☺	☺	?	☺	☺	☻	-	?
Holcomb et al., 2011	☻	☺	☺	☻	?	☻	☺	-	☺
Jacob et al., 2011	?	☺	☺	☻	?	☺	☻	☻	☺
Kim et al., 2011	☺	☺	☺	☺	☺	☻	☺	☻	☺
Montagnana et al., 2011	☺	?	☺	☺	☺	☺	☺	☺	☺
Moore et al., 2008	?	☻	☺	☺	☺	☺	☻	-	☺
Moore et al. 2009	☺	?	☺	☻	☺	☺	☺	☺	☺
Moore et al., 2011	?	☺	☺	☻	☺	☺	☺	☺	☺
Van Gorp et al. 2011	☺	☺	☺	☺	☺	☺	☻	☻	☺

ROMA: Risk of Ovarian Malignancy Algorithm; HE4: Human Epididymis Protein 4; CA125: Cancer Antigen 125.

☺: low risk; ☻: high risk; ?: unclear risk.

### Performance of ROMA for predicting EOC

**Figure 3:**
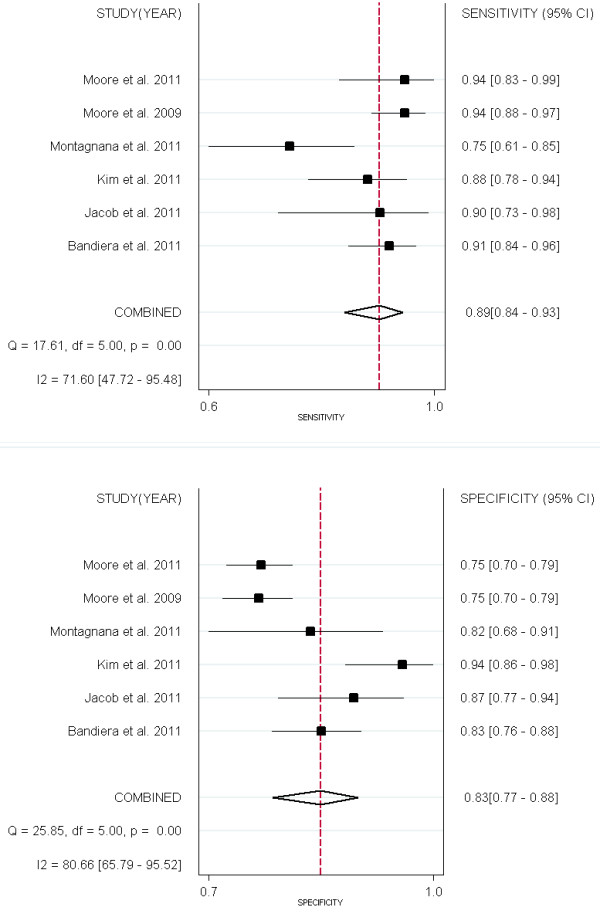
Forest Plots of paired sensitivity and specificity for ROMA.

Forest plots of sensitivity and specificity of ROMA for EOC prediction were shown in Figure3.

 Mean estimates and their 95%CIs were: sensitivity 0.89 (0.84- 0.93), specificity 0.83 (0.77- 0.88) and AUC 0.93 (0.90- 0.95) (Table3). High level of heterogeneity lay in both sensitivity (I^2^ = 71.6%) and specificity (I^2^ = 80.7%).

**Figure 4:**
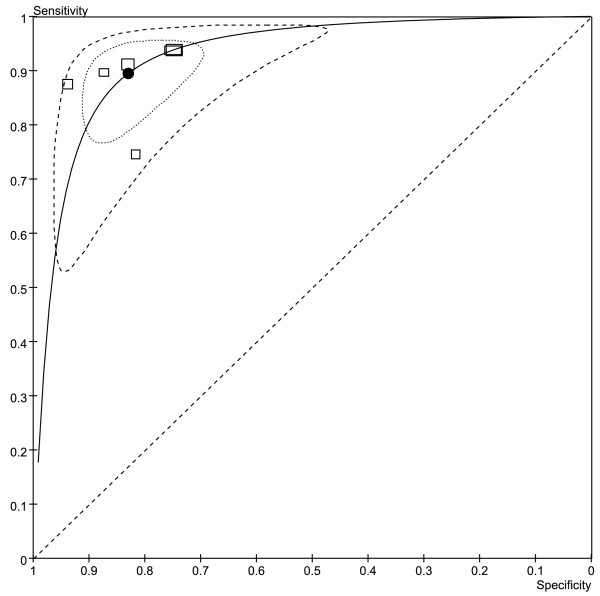
**Hierarchical summary receiver operating characteristic (HSROC) curves and results of bivariate analysis for ROMA to predict EOC.** Results of bivariate analysis: estimates of each studies (the squares), the summary point (solid circle), 95% confidence region (the small ellipse), 95% prediction region (the big ellipse) and HSROC (solid line) were shown. Each study is represented by each square in the meta-analysis. The size of the square indicates the size of each study.

 Threshold effect existed (Spearman correlation coefficient: 0.657, p = 0.156).Thus bivariate model was used to pool estimates. HSROC plots showed the summary estimates of sensitivity and specificity as well as the confidence and prediction regions (Figure4).

Subgroups analysis observed variability in pooled estimates (Table3). We have compared these estimates between subgroups to investigate the performance of ROMA. Across all subgroups, performance (AUCs) of ROMA for EOC detection ranged from 0.88 to 0.97. The ROMA performed better in EOC whole population (AUC: 0.93, 95%CI 0.90- 0.95) than in either premenopausal subgroup (EOC-preM) (AUC: 0.88, 95% CI 0.85- 0.91) or postmenopausal subgroup (EOC-postM) (AUC: 0.89, 95% CI 0.86- 0.92). And the ROMA had better performence in EOC-advanced stage group (AUC: 0.88, 95% CI 0.85- 0.91) than in both EOC whole population and EOC-early stage group (AUC: 0.88, 95% CI 0.83- 0.93). What’s more, the ROMA performed better in EOC population than in OC population (AUC: 0.89, 95% CI 0.87- 0.92).

ROMA had lower sensitivity in premenopausal subgroup (EOC-preM) (0.82, 95%CI 0.67- 0.91) than postmenopausal subgroup (EOC-postM) (0.93, 95%CI 0.89- 0.96). EOC group (0.83, 95% CI 0.77- 0.88) had higher specificity than both EOC-early stage (0.76, 95% CI 0.73- 0.79) and EOC-advanced stage (0.76, 95% CI 0.73- 0.79) groups. ROMA had higher sensitivity in EOC-advanced stage group (0.98, 95%CI: 0.94-1.00) than in EOC whole population (0.90, 95% CI 0.84- 0.93) and EOC-early stage group (0.81, 95% CI 0.71- 0.89). In addition, we found in subgroup method with Concern Regarding Applicability, ROMA had higher specificity in high Concern Regarding Applicability group (EOC-methods High concern) (0.87, 95% CI 0.83- 0.90) than both high Concern Regarding Applicability group (EOC-methods Low concern) (0.75, 95% CI 0.72- 0.78) and EOC whole population. Finally, No differences were found in other summary estimates (except AUC between EOC and OC groups) within EOC, EOC (LMP/BL) and OC groups (Table3).

**Figure 5:**
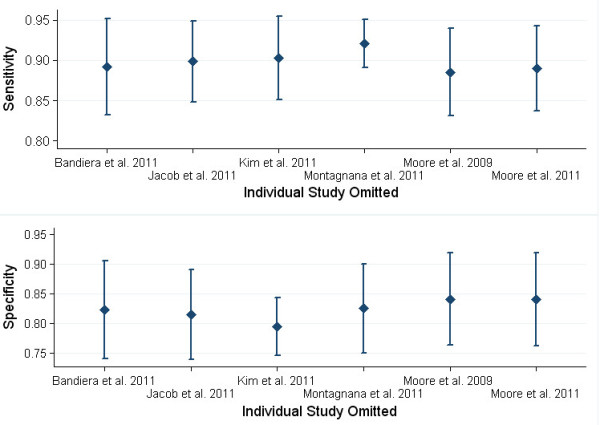
**Influence analysis of individual studies for performance of ROMA to predict EOC.** The meta-analysis was reestimated by omitting each study in turn. The diamonds represented the estimates of the left studies, with their 95% confidence intervals (solid) went through their centers.

The appearance of the Deeks’ funnel plot for ROMA on EOC detection was symmetrical (Additional file 2: Figure S1), and the funnel plot asymmetry test showed little sign of publication bias (regression coefficients was −3.73; p = 0.617). When single study was omitted, the summary estimates (sensitivity, specificity and DOR) were close to those obtained with all eligible studies (Figure5 & Additional file 3: Table S2).

**Table 3:** Summary estimates of ROMA for EOC and OC prediction

**Clinical Settings (study numbers) [analysis model]**	**Mean Sen & (95% CI)**	**Mean Spe & (95% CI)**	**Mean DOR & (95% CI)**	**Mean LR + & (95% CI)**	**Mean LR- & (95% CI)**	**AUC & (95% CI)**
**EOC (n = 6) [bi]**	0.89 (0.84-0.93)	0.83 (0.77-0.88)	41.43 (26.17-65.57)	5.25 (3.85-7.16)	0.13 (0.08-0.20)	0.93 (0.90-0.95)^=^
	[I^2^ =71.6%]	[I^2^ =80.7%]	[I^2^ =44.2%]	[I^2^ =75.7%]	[I^2^ =71.6%]	
**EOC- preM (n = 5) [bi]**	0.82 (0.67-0.91)	0.82 (0.74-0.88)	20.55 (9.70-43.53)	4.50 (3.19-6.36)	0.22 (0.12-0.42)	0.88 (0.85-0.91) ^●^
	[I^2^ =60.1%]	[I^2^ =74.8%]	[I^2^ =43.3%]	[I^2^ =66.0%]	[I^2^ =62.5%]	
**EOC- postM (n = 5) [bi]**	0.93 (0.89-0.96) ^▴^	0.79 (0.73-0.83)	47.27 (27.34-81.73)	4.33 (3.41-5.50)	0.09 (0.06-0.15)	0.89 (0.86-0.92) ^●^
	[I^2^ =51.6%]	[I^2^ =16.2%]	[I^2^ =0.0%]	[I^2^ =15.6%]	[I^2^ =45.8%]	
**EOC- early stage (n = 3) [uni]**	0.81 (0.71-0.89) ^●=^	0.76 (0.73-0.79) ^●^	17.18 (9.08-32.50) ^=^	3.67 (2.56-5.28)	0.24 (0.15-0.38)	0.88 (0.83-0.93)^=^
	[I^2^ =0.0%]	[I^2^ =68.2%]	[I^2^ =0.0%]	[I^2^ =63.4%]	[I^2^ =0.0%]	
**EOC- advanced stage (n = 3) [uni]**	0.98 (0.94-1.00) ^●^	0.76 (0.73-0.79) ^●^	149.08 (47.80-464.95)	4.17 (3.37-5.17)	0.04 (0.01-0.13)	0.97 (0.95-1.00)
	[I^2^ =49.8%]	[I^2^ =68.2%]	[I^2^ =0.0%]	[I^2^ =55.0%]	[I^2^ =28.1%]	
**EOC- methods High concern (n = 3) [uni]**	0.90 (0.85-0.93)	0.87 (0.83-0.90) ^★●^	62.84 (3.25-112.04)	7.29(4.33-12.26)	0.12 (0.08-0.18)	0.95 (0.93-0.97)
	[I^2^ =0.0%]	[I^2^ =67.5%]	[I^2^ =0.0%]	[I^2^ =58.4%]	[I^2^ =0.0%]	
**EOC- methods Low concern (n = 3) [uni]**	0.89 (0.85-0.93)	0.75 (0.72-0.78) ^●^	29.57 (12.85-68.03)	3.74 (3.29-4.25)	0.14 (0.04-0.44)	0.91 (0.86-0.96)
	[I^2^ =85.5%]	[I^2^ =0.0%]	[I^2^ =56.8%]	[I^2^ =0.0%]	[I^2^ =88.2%]	
**EOC (LMP/BL) (n = 3) [uni]**	0.88 (0.84-0.92)	0.77 (0.74-0.80)	33.36 (15.02-74.06)	4.37 (2.88-6.64)	0.15 (0.11-0.20)	0.92 (0.88-0.96)
	[I^2^ =0.0%]	[I^2^ =89.3%]	[I^2^ =66.8%]	[I^2^ =84.6%]	[I^2^ =0.0%]	
**OC (n = 3) [uni]**	0.86 (0.82-0.89)	0.78 (0.75-0.81)	21. 436 (15.28-30.08)	4.11 (3.14-5.38)	0.19 (0.14-0.23)	0.89 (0.87-0.92)^●^
	[I^2^ =0.0%]	[I^2^ =68.9%]	[I^2^ =0.0%]	[I^2^ =61.8%]	[I^2^ =0.0%]	

ROMA: Risk for Ovarian Malignancy Algorithm; EOC: epithelial ovarian cancer; OC: ovarian cancer; Sen: sensitivity; spe: specificity; DOR: diagnostic odds ratio; LR+: positive likelihood ratio; LR-: negative likelihood ratio; CI: confidence interval; bi: bivariate model; uni: univariate model (random effects model); preM: premenopausal; postM: post-menopausal; LMP: low malignant potential tumors; BL: borderline tumors. ^▴^ compared to premenopausal group, ^=^ compared to advanced stage group, ^★^ compared to methods Low concern group, and ^●^ compared to EOC group. Cells labeled with characters have significant difference (p < 0.05) in corresponding estimates with the compared groups. I^2^ was calculated for estimates (sen, spe, DOR and LR±).

## Performance comparison between HE4 and CA125

**Figure 6:**
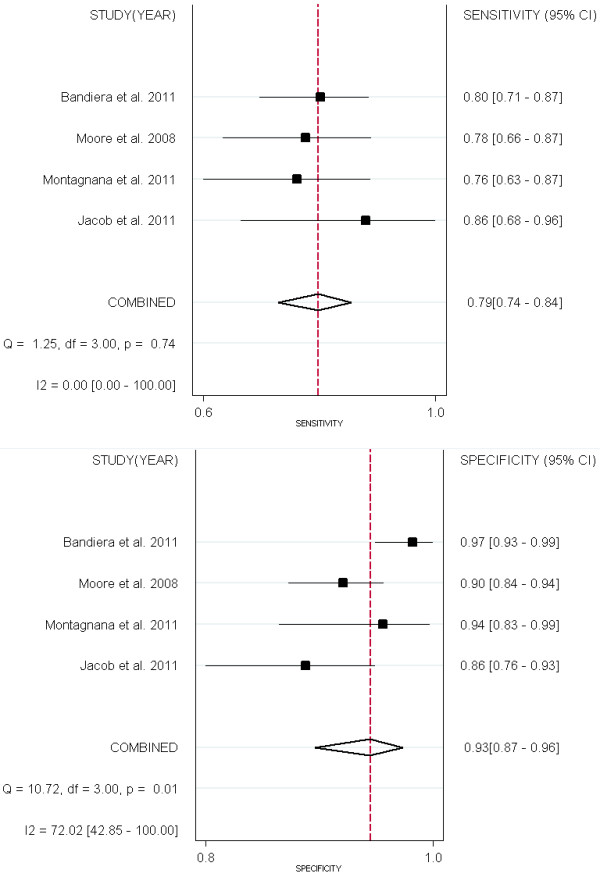
Forest Plots for sensitivity and specificity of HE4 to predict EOC.

**Figure 7:**
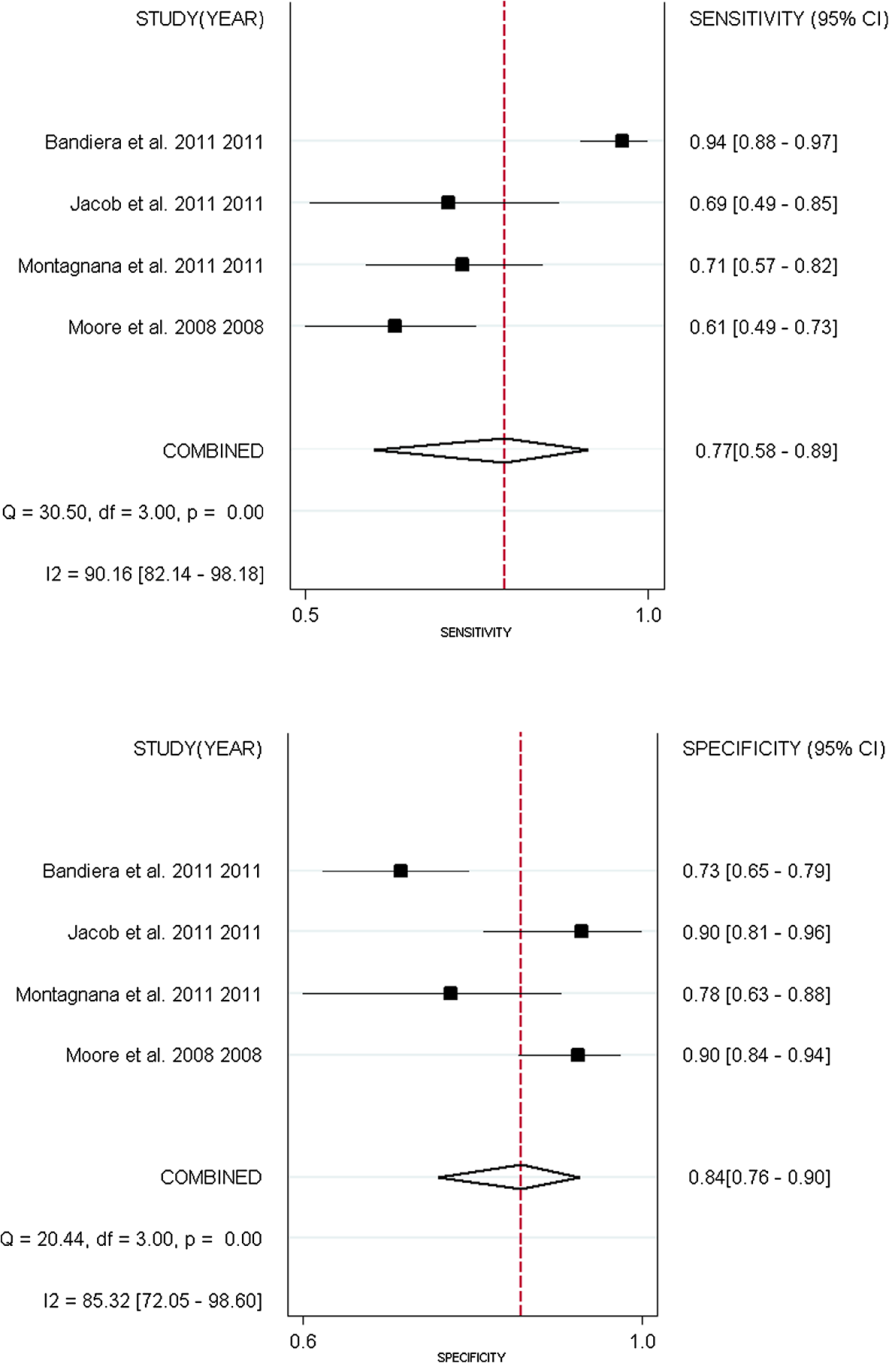
Forest Plots for sensitivity and specificity of CA125 to predict EOC.

**Figure 8:**
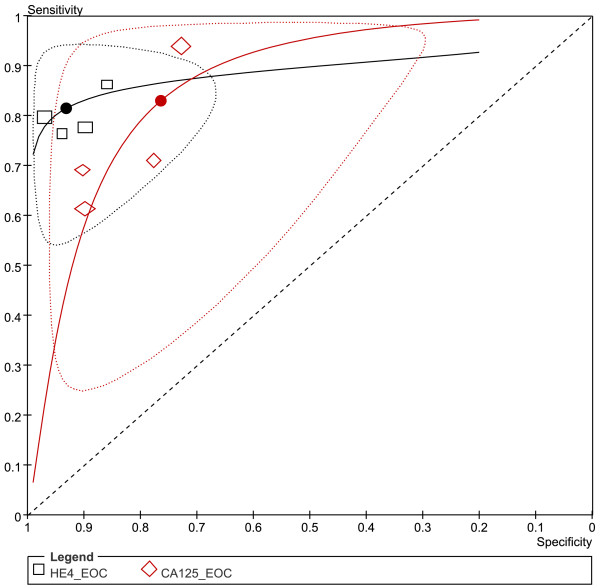
**Hierarchical summary receiver operating characteristic (HSROC) curves and results of bivariate analysis for HE4 and CA125 to predict EOC.** Results of bivariate analysis: estimates of each studies (the squares), the summary point (solid circle), 95% confidence region (the ellipse) and HSROC (solid line) for HE4 (black) and CA125 (red) were shown. Each study is represented by each square in the meta-analysis. The size of the square indicates the size of each study.

**Figure 9:**
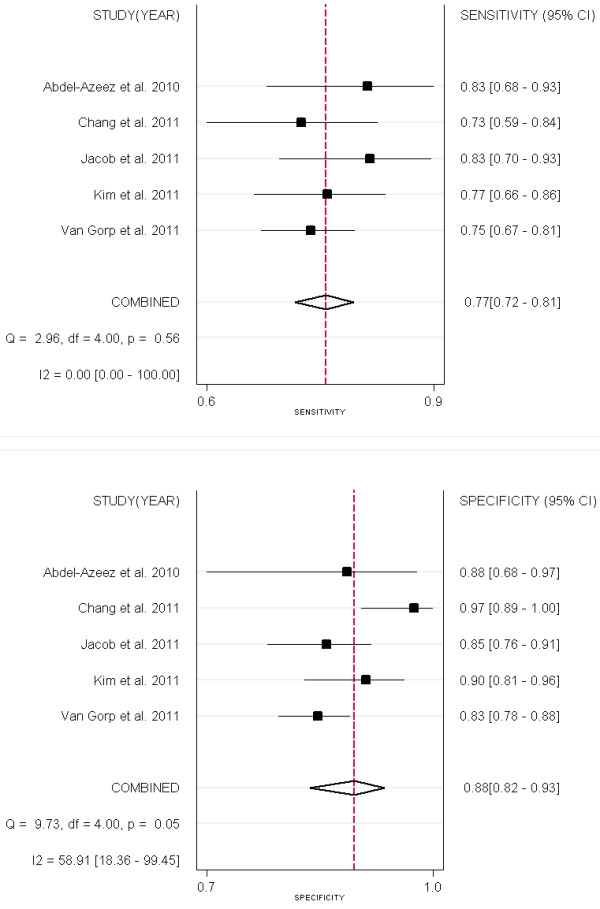
Forest Plots for sensitivity and specificity of HE4 to predict OC

**Figure 10:**
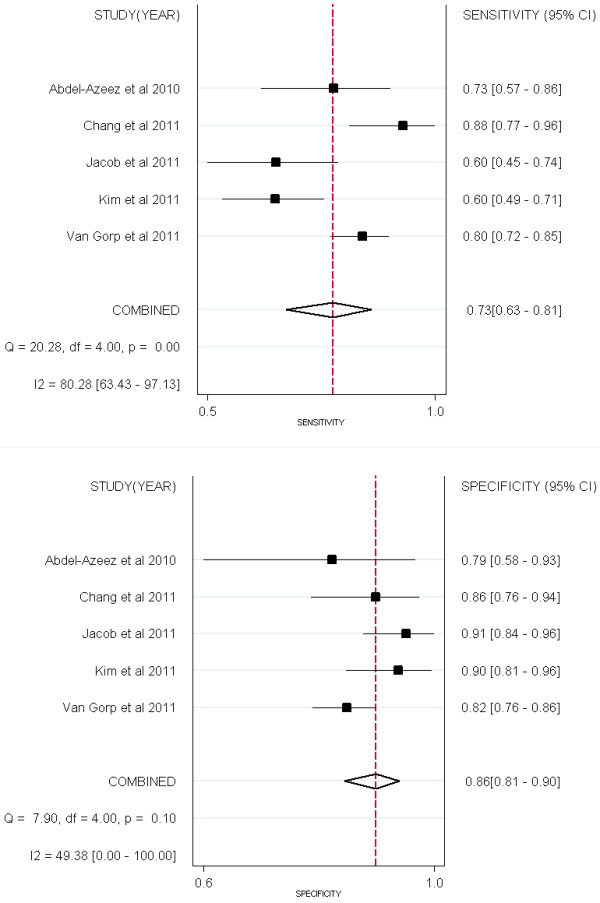
Forest Plots for sensitivity and specificity of CA125 to predict OC.

**Figure 11:**
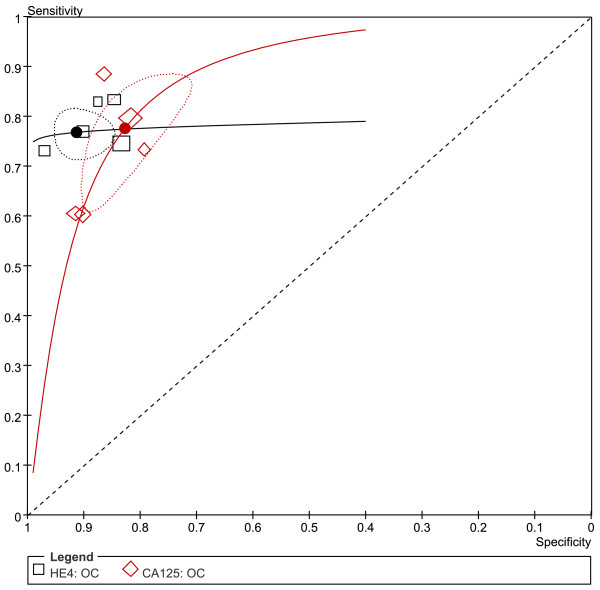
**Hierarchical summary receiver operating characteristic (HSROC) curves and results of bivariate analysis for HE4 and CA125 to predict OC.** Results of bivariate analysis: estimates of each studies (the squares), the summary point (solid circle), 95% confidence region (the ellipse) and HSROC (solid line) for HE4 (black) and CA125 (red) were shown. Each study is represented by each square in the meta-analysis. The size of the square indicates the size of each study.

Four studies [13,15,18,36] compared the performance of HE4 and CA125 for predicting EOC (Figures 6 &7). All the 2 groups (EOC-HE4, EOC-CA125) were analyzed in bivariate model (Figure8). CA125 had a higher AUC than HE4, while a lower specificity than HE4. No significant differences were found within other paired estimates (Table4). Five studies [16,33-36] compared the performance of HE4 and CA125 for predicting OC (Figures 9 &10). All the 2 groups (OC-HE4, OC-CA125) were also analyzed via bivariate model (Figure11). CA125 had a higher AUC than HE4, while no significant differences were found within other paired estimates (Table4).

**Table 4:** Performance comparison between HE4 and CA125 for EOC and OC prediction

**Settings (study numbers) [analysis model]**		**Mean Sen (95% CI)**	**Mean Spe (95% CI)**	**Mean DOR (95% CI)**	**Mean LR+ (95% CI)**	**Mean LR- (95% CI)**	**AUC (95% CI)**
**EOC**	**EOC- HE4 (n = 4) [bi]**	0.79 (0.74-0.84)	0.93 (0.87-0.96)	47.59 (23.87-94.90)	10.64 (5.93-19.10)	0.22 (0.17-0.29)	0.82 (0.78-0.85) ^#^
		[I^2^ =0.0%]	^#^ [I^2^ =72.0%]	[I^2^ =41.3%]	[I^2^ =71.3%]	[I^2^ =0.0%]	
	**EOC-CA125 (n = 4) [bi]**	0.77 (0.58-0.89)	0.84 (0.76-0.90)	18.86 (10.22-31.21)	4.90 (3.63-6.61)	0.27 (0.15-0.50)	0.88 (0.85-0.91)
		[I^2^ =90.2%]	[I^2^ =85.3%]	[I^2^ =57.4%]	[I^2^ =56.2%]	[I^2^ =85.2%]	
**OC**	**OC-HE4 (n = 5) [bi]**	0.77(0.72-0.81)	0.89 (0.82-0.93)	25.37 (14.58-44.14)	6.66 (4.25-10.43)	0.26 (0.22-0.32)	0.79 (0.76-0.83) ^#^
		[I^2^ =0.0%]	[I^2^ =58.9%]	[I^2^ =40.7%]	[I^2^ =50.3%]	[I^2^ = 0.0%]	
	**OC-CA125 (n = 5) [bi]**	0.73 (0.63-0.81)	0.86 (0.81-0.90)	17.12 (11.64-25.19)	5.35 (4.09-7.00)	0.31 (0.23-0.43)	0.89 (0.85-0.91)
		[I^2^ =80.3%]	[I^2^ =49.4%]	[I^2^ =7.8%]	[I^2^ =0.0%]	[I^2^ =75.2%]	

Sen: sensitivity; spe: specificity; DOR: diagnostic odds ratio; LR+: positive likelihood ratio; LR-: negative likelihood ratio; CI: confidence interval; bi: bivariate model; # compared to CA125 with significant difference (p<0.05). All estimates in OC group have no significant difference (p>0.05) with corresponding estimates in EOC group. I^2^ for estimates (sen, spe, DOR and LR±) were calculated.

Studies included also investigated the diagnostic value of HE4 and CA125 in early stage of EOC, as well as distinguishing EOC from benign pelvic mass in premenopausal and postmenopausal women. Because all these settings contained less than 3 studies, we didn’t pool them as subgroups but summarized their sensitivity specificity with forest plots (Additional file 4: Figure S2).

## Performance comparison among ROMA, HE4 and CA125 for EOC prediction

**Figure 12:**
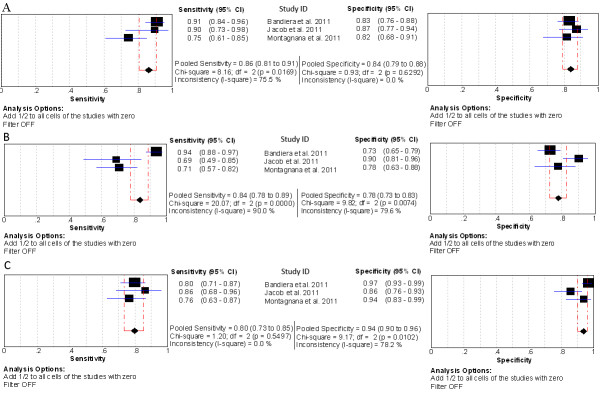
**Forest Plots for sensitivity and specificity comparison among ROMA, HE4 and CA125 to predict EOC.** A: ROMA; B: CA125; C: HE4.

**Figure 13:**
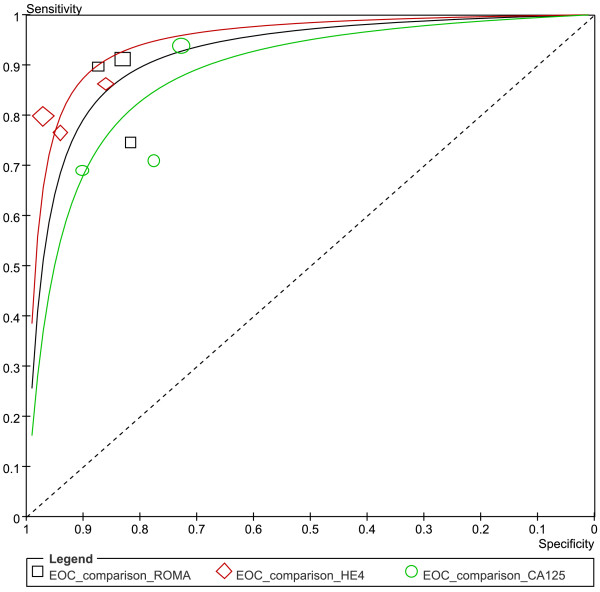
**Summary receiver operating characteristic (SROC) curves for ROMA, HE4 and CA125 to predict EOC.** Results of bivariate analysis: estimates of each studies (the squares) and SROCs (solid line) for ROMA (black), HE4 (red) and CA125 (green) were shown. Each study is represented by each square in the meta-analysis. The size of the square indicates the size of each study. The AUCs and 95% CIs of ROMA, HE4 and CA125 are 0.92 (0.86- 0.97), 0.95 (0.92- 0.98) and 0.88 (0.81- 0.96), respectively.

Three studies evaluated the performance of HE4, CA125 and ROMA for EOC detection (Figure12). All three groups (EOC- ROMA, EOC- HE4 and EOC- CA125) were pooled with univariate model (Figure13 & Table5).

**Table 5:** Summary estimates of comparison among ROMA, HE4 and CA125 for EOC prediction

**Settings (study numbers) [analysis model]**	**Mean Sen (95% CI)**	**Mean Spe (95% CI)**	**Mean DOR (95% CI)**	**Mean LR+ (95% CI)**	**Mean LR- (95% CI)**	**AUC (95% CI)**
EOC- ROMA	0.86 (0.81-0.91)*	0.84 (0.79-0.88) *^#^	32.72 (12.42-86.21)	5.35 (4.09-7.01)	0.17 (0.07-0.40)	0.92 (0.86-0.97)
(n = 3) [uni]	[I^2^ =75.5%]	[I^2^ =0.0%]	[I^2^ =64.4%]	[I^2^ =0.0%]	[I^2^ =80.5%]	
EOC-HE4	0.80 (0.73-0.85)	0.94 (0.90-0.96)	67.99 (31.97-144.60)	12.21 (4.25-35.11)	0.22 (0.17-0.29)	0.95 (0.92-0.98)
(n = 3) [uni]	[I^2^ =0.0%]	[I^2^ =78.2%]	[I^2^ =19.2%]	[I^2^ =79.0%]	[I^2^ =0.0%]	
EOC-CA125	0.84 (0.78-0.89)	0.78 (0.73-0.83) *	19.15 (7.26-50.53)	3.81 (2.66-5.46)	0.23 (0.09-0.58)	0.88 (0.81-0.96)
(n = 3) [uni]	[I^2^ =90.0%]	[I^2^ =79.6%]	[I^2^ =68.8%]	[I^2^ =41.4%]	[I^2^ =87.6%]	

Sen: sensitivity; spe: specificity; DOR: diagnostic odds ratio; LR+: positive likelihood ratio; LR-: negative likelihood ratio; CI: confidence interval; uni: univariate model (random effects model); * compared to HE4; ^#^ compared to CA125. Cells labeled with characters have significant difference (p < 0.05) in corresponding estimates with the compared groups. I^2^ was calculated for estimates (sen, spe, DOR and LR±).

Among the three tests, HE4 had the highest specificity (0.94, 95% CI: 0.90-0.96), but a lower sensitivity (0.80, 95% CI: 0.73-0.85) than ROMA (0.86, 95% CI: 0.81-0.91). The ROMA had a higher specificity (0.84, 95% CI: 0.79-0.88) than CA125 (0.78, 95% CI: 0.73-0.83). Meanwhile no differences were found between CA125 and HE4, as well as between CA125 and ROMA in their summary sensitivity. The DOR, LR ± and AUC values were similar among the three tests (Table5).

## Discussion

### Summary of main results

Our results found that, first, ROMA could help distinguish EOC from benign pelvic mass with a high diagnostic accuracy (AUC: 0.93). The ROMA has high sensitivity to predict advanced stage EOC than early stage EOC and in postmenopausal women than in premenopausal women. Second, although HE4 has higher specificity than CA125 for EOC monitoring, CA125 has better diagnosis accuracy (higher AUC) than HE4 for EOC or OC prediction. This is based on the results of 4 studies that compare HE4 and CA125 within the same population. Third, based on the results of comparison of HE4, CA125 and ROMA in the same population, the overall performance (AUC) of the three tests for EOC prediction are similar. ROMA is less specific but more sensitive than HE4, while both ROMA and HE4 are more specific than CA125 for EOC monitoring.

All studies included were subjected to close scrutiny with the QUADAS-2 tool, resulting in high quality across the items. Heterogeneity often existed in diagnostic meta-analysis [38], and mainly resulted from characteristics of the study population, variations in the study design, different statistical methods, and different covariates [39]. Within-study quality were highly concerned in this meta-analysis. Both high level of heterogeneity in sensitivity and specificity were found for ROMA test. The existence of threshold effect might partially explain the heterogeneity. Analysis of subgroups (EOC-methods high concern and EOC-methods low concern) found the EOC-methods High concern group had higher specificity than both EOC-methods Low concern group and EOC group.

In the current paper, only three studies evaluated the diagnostic value of ROMA at early stage of EOC. The early stage ovarian cancer usually presented non-specific clinical manifestation, and the FIGO staging by surgery often resulted in low prevalence of early stage EOC. So future clinical investigations will be promising and expectant to be prospective studies recruiting enough patients with early stage EOC.

We analyzed the predictive value of ROMA for patients with EOC, EOC(LMP/BL) and ovarian cancer. No differences were found in all summary estimates (except AUC between EOC and OC groups) of EOC, EOC (LMP/BL) and OC groups. Although EOC accounted for 90% of ovarian cancer, we didn’t think ROMA could be expanded to predict ovarian cancer, for both HE4 and CA125 were biomarkers of epithelial ovarian cancer[2,11].

Cut-off values were variable for HE4 (70-150pM) and ROMA (preM: 7.4-13.1%; postM: 10.9-27.7%), but consistent for CA125 (35U/mL) across studies. Among the studies included, only one study[15] used specific cut-off values for premenopausal (70pM) and postmenopausal women (140pM). Studies found that HE4 levels in healthy subjects were associated with age [40,41]. So it would be essential to define a specific normal range and cut-off value for premenopausal and postmenopausal women respectively. For other two predictors ROMA and CA125, it would also be indispensable for each center to define their normal ranges and cut-off values.

### Strengths and weaknesses

Except employing a comprehensive search strategy, strict inclusion criteria and sound analysis protocol, strengths of this paper also contain that only studies investigating both the two tests (HE4 and CA125) or all three tests (HE4, CA125 and ROMA) in a same population have been included in tests comparisons. The latter makes sure that the comparison takes place between studies under the same or similar population background, thus reduces the heterogeneity between studies [42].

The main limitations are: (1) unable to gain the unpublished paper. (2) Study number might be small. We believe that reliability of the meta-analysis are majorly dependent on the quality of studies included. (3) The diagnostic value of ROMA, HE4 and CA125 in early stage EOC have not been convincingly analyzed.

### Conclusions

ROMA can help distinguish EOC from benign pelvic mass. ROMA is less specific but more sensitive than HE4. Both ROMA and HE4 are more specific than CA125 for EOC prediction. CA125 has better diagnosis accuracy than HE4 for EOC and OC prediction. ROMA is promising predictor to replace CA125, but its utilization requires further exploration.

### Abbreviations

ROMA, Risk for ovarian malignancy algorithm; HE4, Human epididymis protein 4; EOC, Epithelial ovarian cancer; OC, Ovarian cancer; CA125, Cancer antigen 125; QUADAS-2, Quality assessment of diagnostic accuracy studies-2; DOR, Diagnostic odds ratio; LR±, Positive and negative likelihood ratio; AUC, Area under receiver operating characteristic-curve; CI, Confidence interval; FDA, Food and drug administration; EIA, Enzyme immunoassay; MOOSE, Meta-analysis of observational studies in epidemiology; FIGO, International federation of gynecology and obsterics; TPR, True positive rate; FPR, False positive rate; LMP, Low malignant potential tumors; BL, Borderline tumors; HSROC, Hierarchical summary receiver operating characteristic curves; CMIA, Chemiluminescent microparticle immunoassay; RIA, Radioimmunoassay; CLEIA, Chemilumenscence enzyme immunoassay; ECLIA, Electrochemilumenscence immunoassay.

### Competing interests

Lili Yu declared a grant (NO. 81070505) from National Natural Science Foundation of China. For other authors none were declared.

### Authors’ contributions

FKL conceived the study, searched databases, extracted the data, performed the statistical analysis and drafted the manuscript. RXT selected the trials, extracted the data and helped draft the manuscript. KC and WPL participated in the selection of trials and the statistical analysis. FW searched databases, evaluated the studies included and revised the manuscript critically. SLD evaluated the studies included, participated in the coordination and revised the manuscript critically. LLY participated in the design, selected the trials and revised the manuscript critically. MC conceived of the study together with FKL, participated in the design and helped draft the manuscript. All authors read and approved the final manuscript.

### Links

1 What are the key statistics about ovarian cancer? American Cancer Society Web site. [http://www.cancer.org/Cancer/OvarianCancer/DetailedGuide/ovarian-cancer-key-statistics]

2 Diagnostic Test Accuracy Working Group*.* Handbook for DTA Reviews. Cochrane Collaboration Web site. [http://srdta.cochrane.org/handbook-dta-reviews].

3 International Federation of Gynecology and Obstetrics FIGO website [http://www.figo.org/]

## Pre-publication history

The pre-publication history for this paper can be accessed here:

http://www.biomedcentral.com/1471-2407/12/258/prepub

## Supplementary Material

Additional file 1 **Table S1. ** Searching strategies. Click here for file

Additional file 2 **Figure S1. **Deeks’ funnel plot for ROMA. Click here for file

Additional file 3 **Table S2. **Influence analysis of individual studies for diagnostic performance of ROMA. Estimates were pooled by bivariate model. Excluding any individual study only a small change were resulted in the sensitivity (sen), specificity (spe) or diagnostic odds ratio (DOR) compared with all eligible studies. All differences were not significant (p > 0.05). Click here for file

Additional file 4 **Figure S2. **Forest Plots for comparison between HE4 and CA125 for premenopausal and postmenopausal women and early stage EOC groups. Click here for file

## References

[B1] Brown PO Palmer C The preclinical natural history of serous ovarian cancer: defining the target for early detection PLoS Med 2009 6 e1000114 10.1371/journal.pmed.1000114 19636370 PMC2711307

[B2] Davis HM Zurawski VR Bast RC Klug TL Characterization of the CA 125 antigen associated with human epithelial ovarian carcinomas Cancer Res 1986 46 6143 6148 2430690

[B3] Bast RC Badgwell D Lu Z Marquez R Rosen D Liu J Baggerly KA Atkinson EN Skates S Zhang Z New tumor markers: CA125 and beyond Int J Gynecol Cancer 2005 15 274 281 10.1111/j.1525-1438.2005.00441.x 16343244

[B4] Niloff JM Klug TL Schaetzl E Zurawski VR Knapp RC Bast RC Elevation of serum CA125 in carcinomas of the fallopian tube, endometrium, and endocervix Am J Obstet Gynecol 1984 148 1057 1058 6201072 10.1016/s0002-9378(84)90444-7

[B5] Park Y Lee JH Hong DJ Lee EY Kim HS Diagnostic performances of HE4 and CA125 for the detection of ovarian cancer from patients with various gynecologic and non-gynecologic diseases Clin Biochem 2011 44 884 888 10.1016/j.clinbiochem.2011.04.011 21549107

[B6] Yurkovetsky Z Skates S Lomakin A Nolen B Pulsipher T Modugno F Marks J Godwin A Gorelik E Jacobs I Development of a multimarker assay for early detection of ovarian cancer J Clin Oncol 2010 28 2159 2166 10.1200/JCO.2008.19.2484 20368574 PMC2860434

[B7] Cree I Improved blood tests for cancer screening: general or specific? BMC Cancer 2011 11 499 10.1186/1471-2407-11-499 22128772 PMC3285105

[B8] Kulasingam V Pavlou MP Diamandis EP Integrating high-throughput technologies in the quest for effective biomarkers for ovarian cancer Nat Rev Cancer 2010 10 371 378 10.1038/nrc2831 20383179

[B9] Lu R Sun M Feng J Gao X Guo L Myofibrillogenesis regulator 1 (MR-1) is a novel biomarker and potential therapeutic target for human ovarian cancer BMC Cancer 2011 11 270 10.1186/1471-2407-11-270 21702971 PMC3132198

[B10] Kothandaraman N Bajic VB Brendan PN Huak CY Keow PB Razvi K Salto-Tellez M Choolani M E2F5 status significantly improves malignancy diagnosis of epithelial ovarian cancer BMC Cancer 2010 10 64 10.1186/1471-2407-10-64 20181230 PMC2841139

[B11] Helistrom I Raycraft J Hayden-Ledbetter M Ledbetter JA Schummer M McIntosh M Drescher C Urban N Hellstrom KE The HE4 (WFDC2) protein is a biomarker for ovarian carcinoma Cancer Res 2003 63 3695 3700 12839961

[B12] Drapkin R von Horsten HH Lin Y Mok SC Crum CP Welch WR Hecht JL Human epididymis protein 4 (HE4) is a secreted glycoprotein that is overexpressed by serous and endometrioid ovarian carcinomas Cancer Res 2005 65 2162 2169 10.1158/0008-5472.CAN-04-3924 15781627

[B13] Moore RG Brown AK Miller MC Skates S Allard WJ Verch T Steinhoff M Messerlian G DiSilvestro P Granai CO Bast RC The use of multiple novel tumor biomarkers for the detection of ovarian carcinoma in patients with a pelvic mass Gynecol Oncol 2008 108 402 408 10.1016/j.ygyno.2007.10.017 18061248

[B14] Holcomb K Vucetic Z Miller MC Knapp RC Human epididymis protein 4 offers superior specificity in the differentiation of benign and malignant adnexal masses in premenopausal women Am J Obstet Gynecol 2011 205 358 e351 e356 10.1016/j.ajog.2011.05.017 21722869

[B15] Bandiera E Romani C Specchia C Zanotti L Galli C Ruggeri G Tognon G Bignotti E Tassi RA Odicino F Serum human epididymis protein 4 and risk for ovarian malignancy algorithm as new diagnostic and prognostic tools for epithelial ovarian cancer management Cancer Epidemiol Biomarkers Prev 2011 20 2496 2506 10.1158/1055-9965.EPI-11-0635 22028406 PMC3237732

[B16] Van Gorp T Cadron I Despierre E Daemen A Leunen K Amant F Timmerman D De Moor B Vergote I HE4 and CA125 as a diagnostic test in ovarian cancer: prospective validation of the risk of ovarian malignancy algorithm Br J Cancer 2011 104 863 870 10.1038/sj.bjc.6606092 21304524 PMC3048204

[B17] Moore RG McMeekin DS Brown AK DiSilvestro P Miller MC Allard WJ Gajewski W Kurman R Bast RC Skates SJ A novel multiple marker bioassay utilizing HE4 and CA125 for the prediction of ovarian cancer in patients with a pelvic mass Gynecol Oncol 2009 112 40 46 10.1016/j.ygyno.2008.08.031 18851871 PMC3594094

[B18] Montagnana M Danese E Ruzzenente O Bresciani V Nuzzo T Gelati M Salvagno GL Franchi M Lippi G Guidi GC The ROMA (Risk of Ovarian Malignancy Algorithm) for estimating the risk of epithelial ovarian cancer in women presenting with pelvic mass: is it really useful? Clin Chem Lab Med 2011 49 521 525 21288178 10.1515/CCLM.2011.075

[B19] Stroup DF Berlin JA Morton SC Olkin I Williamson GD Rennie D Moher D Becker BJ Sipe TA Thacker SB Meta-analysis of observational studies in epidemiology: a proposal for reporting. Meta-analysis Of Observational Studies in Epidemiology (MOOSE) group JAMA 2000 283 2008 2012 10.1001/jama.283.15.2008 10789670

[B20] Lijmer JG Mol BW Heisterkamp S Bonsel GJ Prins MH van der Meulen JHP Bossuyt PMM Empirical evidence of design-related bias in studies of diagnostic tests JAMA 1999 282 1061 1066 10.1001/jama.282.11.1061 10493205

[B21] Benedet JL Pecorelli S Why cancer staging? FIGO 26th Annual Report on the Results of Treatment in Gynecological Cancer Int J Gynaecol Obstet 2006 95 Suppl 1 S3 17161166 10.1016/S0020-7292(06)60026-X

[B22] Whiting PF Rutjes AWS Westwood ME Mallett S Deeks JJ Reitsma JB Leeflang MMG Sterne JAC Bossuyt PMM Group tQ-: QUADAS-2: a revised tool for the quality assessment of diagnostic accuracy studies Ann Intern Med 2011 155 529 536 22007046 10.7326/0003-4819-155-8-201110180-00009

[B23] Whiting P Harbord R Kleijnen J No role for quality scores in systematic reviews of diagnostic accuracy studies BMC Med Res Methodol 2005 5 19 10.1186/1471-2288-5-19 15918898 PMC1184082

[B24] Chappell FM Raab GM Wardlaw JM When are summary ROC curves appropriate for diagnostic meta-analyses? Stat Med 2009 28 2653 2668 10.1002/sim.3631 19591118

[B25] Rutter CM Gatsonis CA A hierarchical regression approach to meta-analysis of diagnostic test accuracy evaluations Stat Med 2001 20 2865 2884 10.1002/sim.942 11568945

[B26] Reitsma JB Glas AS Rutjes AW Scholten RJ Bossuyt PM Zwinderman AH Bivariate analysis of sensitivity and specificity produces informative summary measures in diagnostic reviews J Clin Epidemiol 2005 58 982 990 10.1016/j.jclinepi.2005.02.022 16168343

[B27] Higgins JP Thompson SG Deeks JJ Altman DG Measuring inconsistency in meta-analyses BMJ 2003 327 557 560 10.1136/bmj.327.7414.557 12958120 PMC192859

[B28] Zamora J Abraira V Muriel A Khan K Coomarasamy A Meta-DiSc: a software for meta-analysis of test accuracy data BMC Med Res Methodol 2006 6 31 10.1186/1471-2288-6-31 16836745 PMC1552081

[B29] Leeflang MMG Deeks JJ Gatsonis C Bossuyt PMM Group obotCDTAW: Systematic reviews of diagnostic test accuracy Ann Intern Med 2008 149 889 897 19075208 10.7326/0003-4819-149-12-200812160-00008 PMC2956514

[B30] Deeks JJ Macaskill P Irwig L The performance of tests of publication bias and other sample size effects in systematic reviews of diagnostic test accuracy was assessed J Clin Epidemiol 2005 58 882 893 10.1016/j.jclinepi.2005.01.016 16085191

[B31] Dwamena BAmidas: Computational and Graphical Routines for Meta-analytical Integration of Diagnostic Accuracy Studies in Stata 2007 Division of Nuclear Medicine, Department of Radiology, University of Michigan Medical School, Ann Arbor

[B32] Roger M Harbord PW metandi: Meta-analysis of diagnostic accuracy using hierarchical logistic regression Stata J 2009 9 19

[B33] Abdel-Azeez HA Labib HA Sharaf SM Refaie AN HE4 and mesothelin: novel biomarkers of ovarian carcinoma in patients with pelvic masses Asian Pac J Cancer Prev 2010 11 111 116 20593939

[B34] Kim YM Whang DH Park J Kim SH Lee SW Park HA Ha M Choi KH Evaluation of the accuracy of serum human epididymis protein 4 in combination with CA125 for detecting ovarian cancer: a prospective case–control study in a Korean population Clin Chem Lab Med 2011 49 527 534 21320028 10.1515/CCLM.2011.085

[B35] Chang X Ye X Dong L Cheng H Cheng Y Zhu L Liao Q Zhao Y Tian L Fu T Human epididymis protein 4 (HE4) as a serum tumor biomarker in patients with ovarian carcinoma Int J Gynecol Cancer 2011 21 852 858 10.1097/IGC.0b013e31821a3726 21633297

[B36] Jacob F Meier M Caduff R Goldstein D Pochechueva T Hacker N Fink D Heinzelmann-Schwarz V No benefit from combining HE4 and CA125 as ovarian tumor markers in a clinical setting Gynecol Oncol 2011 121 487 491 10.1016/j.ygyno.2011.02.022 21420727

[B37] Moore RG Miller MC Disilvestro P Landrum LM Gajewski W Ball JJ Skates SJ Evaluation of the diagnostic accuracy of the risk of ovarian malignancy algorithm in women with a pelvic mass Obstet Gynecol 2011 118 280 288 10.1097/AOG.0b013e318224fce2 21775843 PMC3594110

[B38] Harbord RM Deeks JJ Egger M Whiting P Sterne JA A unification of models for meta-analysis of diagnostic accuracy studies Biostatistics 2007 8 239 251 16698768 10.1093/biostatistics/kxl004

[B39] Dinnes J Deeks J Kirby J Roderick P A methodological review of how heterogeneity has been examined in systematic reviews of diagnostic test accuracy Health Technol Assess 2005 9 iii 1 113 10.3310/hta9120 15774235

[B40] Bolstad N Oijordsbakken M Nustad K Bjerner J Human epididymis protein 4 reference limits and natural variation in a Nordic reference population Tumour Biol 2012 33 141 148 10.1007/s13277-011-0256-4 22105734 PMC3235278

[B41] Galgano MT Hampton GM Frierson HF Comprehensive analysis of HE4 expression in normal and malignant human tissues Mod Pathol 2006 19 847 853 16607372 10.1038/modpathol.3800612

[B42] Zhou Y Yin X Ying J Zhang B Golgi protein 73 versus alpha-fetoprotein as a biomarker for hepatocellular carcinoma: a diagnostic meta- analysis BMC Cancer 2012 12 17 10.1186/1471-2407-12-17 22244200 PMC3292967

