# Supplementary material for: Does risk for ovarian malignancy algorithm excel human epididymis protein 4 and ca125 in predicting epithelial ovarian cancer: A meta-analysis
Source: BMC Cancer. 2012 Jun 19;12:258. doi: 10.1186/1471-2407-12-258 (PMC3443004; doi:10.1186/1471-2407-12-258)
Supplement: Additional file 1 — Table S1. Searching strategies. [file 1471-2407-12-258-S1.doc]

**Table S1 PubMed literature search strategies.**

| **Search** | **Queries** |
| --- | --- |
| #1 | human epididymis protein 4[MeSH Terms] OR "human epididymis protein 4" OR "human epididymal protein 4" OR he4 OR "he 4" |
| #2 | epididymal secretory protein[MeSH Terms] OR "epididymal secretory protein" OR "epididymal secretory proteins" OR "epididymis secretory protein" OR "epididymis secretory proteins" |
| #3 | wap four disulfide core domain 2[MeSH Terms] OR "wap four disulfide core domain 2" OR "wfdc 2" OR wfdc2 |
| #4 | #1 OR #2 OR #3 |
| #5 | ca 125 antigen[MeSH Terms] OR "ca 125 antigen" OR ca125 OR "ca 125" OR "ca 12 5" OR "ca12 5" OR "cancer antigen 125" OR "cancer antigens 125" OR "mucin 16, cell surface associated" OR MUC16 OR "muc 16" |
| #6 | ovary cancer[MeSH Terms] OR "ovary cancer" or "malignant ovary tumor" |
| #7 | #5 OR #6 |
| #8 | "sensitivity and specificity"[mh] OR sensitiv*[tiab] OR diagnosis[mh:noexp] OR diagnosis, differential[mh:noexp] OR diagnosis[sh] OR diagnos*[tiab] OR predictive value of tests[mh] OR reproducibility of results[mh] |
| #9 | #4 AND #7 AND #8 |

Searching ended on 22th December 2011.
