# Supplementary figures and images for: Does risk for ovarian malignancy algorithm excel human epididymis protein 4 and ca125 in predicting epithelial ovarian cancer: A meta-analysis
Source: BMC Cancer. 2012 Jun 19;12:258. doi: 10.1186/1471-2407-12-258 (PMC3443004; doi:10.1186/1471-2407-12-258)

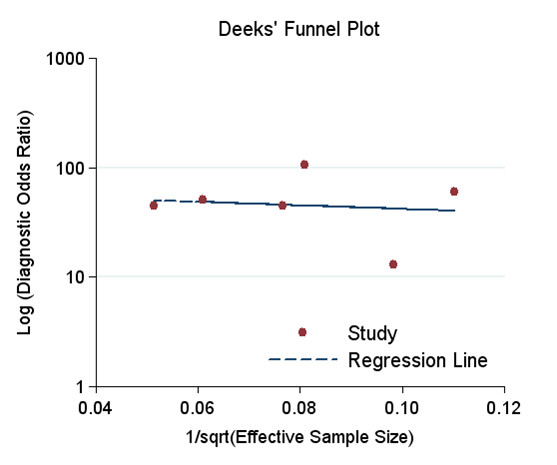

Supplement: Additional file 2 — Figure S1. Deeks’ funnel plot for ROMA. [file 1471-2407-12-258-S2.jpeg]

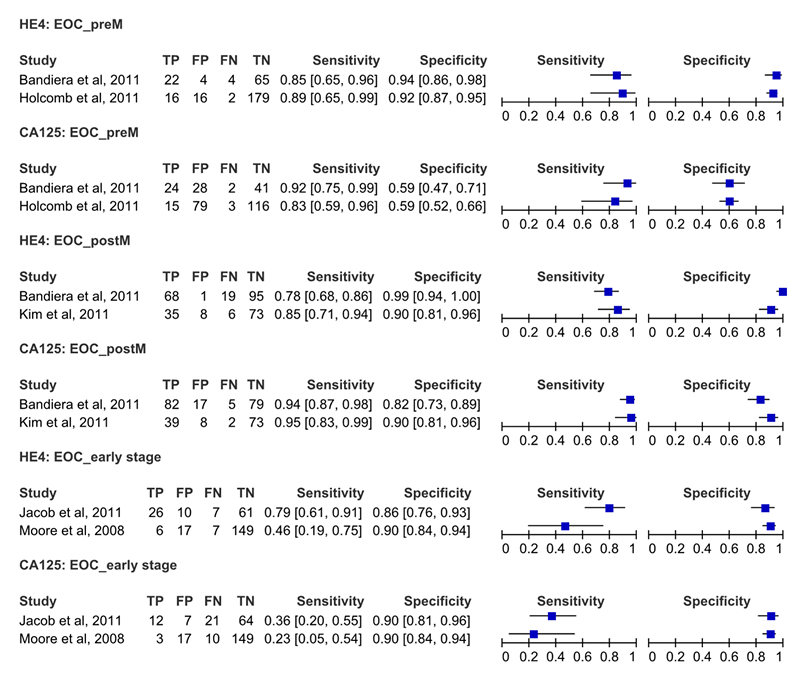

Supplement: Additional file 4 — Figure S2. Forest Plots for comparison between HE4 and CA125 for premenopausal and postmenopausal women and early stage EOC groups. [file 1471-2407-12-258-S4.jpeg]
