# Supplementary material for: Does risk for ovarian malignancy algorithm excel human epididymis protein 4 and ca125 in predicting epithelial ovarian cancer: A meta-analysis
Source: BMC Cancer. 2012 Jun 19;12:258. doi: 10.1186/1471-2407-12-258 (PMC3443004; doi:10.1186/1471-2407-12-258)
Supplement: Additional file 3 — Table S2. Influence analysis of individual studies for diagnostic performance of ROMA. Estimates were pooled by bivariate model. Excluding any individual study only a small change were resulted in the sensitivity (sen), specificity (spe) or diagnostic odds ratio (DOR) compared with all eligible studies. All differences were not significant (p > 0.05). [file 1471-2407-12-258-S3.doc]

**Table S2. Influence analysis of individual studies for diagnostic performance of ROMA**

| Studies (omitted) | Pooled sen (95%CI) | Pooled spe (95%CI) | Pooled DOR (95%CI) | Comparison with no omitting (p-value) |
| --- | --- | --- | --- | --- |
| None of 6 studies | 0.895 (0.837-0.934) | 0.830 (0.765-0.879) | 41.426 (26.173-65.568) | --- |
| Bandiera et al. 2011 | 0.890 (0.816-0.937) | 0.831 (0.750-0.890) | 40.038 (22.109-72.505) | All (Sen Spe DOR) p >0.05 |
| Jacob et al. 2011 | 0.896 (0.830-0.939) | 0.822 (0.749-0.877) | 39.786 (23.999-65.958) | All (Sen Spe DOR) p >0.05 |
| Kim et al. 2011 | 0.899 (0.827-0.942) | 0.799 (0.744-0.844) | 35.157 (21.189-58.331) | All (Sen Spe DOR) p >0.05 |
| Montagnana et al. 2011 | 0.915 (0.875-0.943) | 0.831 (0.750-0.890) | 52.795 (32.973-85.108) | All (Sen Spe DOR) p >0.05 |
| Moore et al. 2009 | 0.881 (0.813-0.927) | 0.846 (0.776-0.897) | 40.703 (21.946-75.493) | All (Sen Spe DOR) p >0.05 |
| Moore et al. 2011 | 0.885 (0.820-0.929) | 0.846 (0.778-0.896) | 42.404 (24.483-73.442) | All (Sen Spe DOR) p >0.05 |

Estimates were pooled by bivariate model. Excluding any individual study only a small change were resulted in the sensitivity (sen), specificity (spe) or diagnostic odds ratio (DOR) compared with all eligible studies. All differences were not signiﬁcant (p>0.05).
